# The genome sequence of the least weasel, *Mustela nivalis* Linnaeus, 1766 (Carnivora: Mustelidae)

**DOI:** 10.12688/wellcomeopenres.24862.1

**Published:** 2025-09-08

**Authors:** Michelle F. O’Brien, Rosa Lopez Colom, Natural History Museum Genome Acquisition Lab, Wellcome Sanger Institute Tree of Life Management, Samples and Laboratory team, Wellcome Sanger Institute Scientific Operations: Sequencing Operations, Wellcome Sanger Institute Tree of Life Core Informatics team, Tree of Life Core Informatics collective, Darwin Tree of Life Consortium

**Affiliations:** 1 Wildfowl & Wetlands Trust, Slimbridge, Gloucestershire, England, UK

**Keywords:** Mustela nivalis; least weasel; genome sequence; chromosomal; Carnivora

## Abstract

We present a genome assembly from a male *Mustela nivalis* (least weasel; Chordata; Mammalia; Carnivora; Mustelidae). The assembly contains two haplotypes with total lengths of 3 437.86 megabases and 2 835.05 megabases. Haplotype 1 is scaffolded into 22 chromosomal pseudomolecules, including the X and Y sex chromosomes. Haplotype 2 was assembled to scaffold level. The mitochondrial genome has also been assembled, with a length of 16.49 kilobases.

## Species taxonomy

Eukaryota; Opisthokonta; Metazoa; Eumetazoa; Bilateria; Deuterostomia; Chordata; Craniata; Vertebrata; Gnathostomata; Teleostomi; Euteleostomi; Sarcopterygii; Dipnotetrapodomorpha; Tetrapoda; Amniota; Mammalia; Theria; Eutheria; Boreoeutheria; Laurasiatheria; Carnivora; Caniformia; Musteloidea; Mustelidae; Mustelinae; *Mustela*; *Mustela nivalis* Linnaeus, 1766 (NCBI:txid36239)

## Background

The Least Weasel ( *Mustela nivalis*) is a small mustelid with chestnut coloured fur dorsally and white fur ventrally. It has a blunt nose, small eyes and ears situated towards the back of the head. This species is the smallest member of the Order Carnivora. Their body is long and slender with a short, chestnut brown tail and usually measures 20–30 cm in length. Their limbs are short and end in clawed toes. This species is usually solitary in nature, mating in early spring with litters of 4 to 6 kits born between April and August. Weaning takes place at 3 to 4 months of age and the young are usually independent in 2 to 3 months ( Vass & Bende, 2024; Woodland Trust, 2025). The lifespan of this species in the wild is usually approximately 2 years (only 10% survive beyond this) ( Mammal Society, 2024), although the oldest captive specimen was 9.1 years old when it died ( Weigl, 2005).

The Least Weasel shows a circumboreal Holarctic distribution including most of Europe, North Asia and northern North America ( Abramov & Baryshnikov, 2000; Sheffield & King, 1994). Its European distribution includes Britain but not Ireland. In Asia it is found from Northern Mongolia, Korea and Northern China to Lebanon, Iran and Afghanistan ( Abramov & Baryshnikov, 2000; Bannikov, 1954; Dulamtseren, 1970; Habibi, 2003; Wang, 2003; Won & Smith, 1999). It is also found on a number of islands including those of Japan ( Abe *et al.*, 2005), Russia ( Abramov & Baryshnikov, 2000) and Taiwan ( Abramov, 2006). Its population in North Africa (Morocco, Algeria and Tunisia) has been thought to be native but it has also been postulated that they may have been introduced ( Lebarbenchon *et al.*, 2010). It has been introduced to a number of Mediterranean islands, the Azores Islands, São Tomé off West Africa, and New Zealand ( Dobson, 1998; Lebarbenchon *et al.*, 2010; Sheffield & King, 1994).

This species has been reported from sea level up to at least 3 860 m. It is found in a wide range of habitats which include forest, farmland and cultivated fields, grassy fields and meadows, riparian woodland, hedgerows, alpine meadows and forest, scrub, steppe, semidesert, prairies, and coastal dunes ( Pulliainen, 1999; Sheffield & King, 1994). It builds its den in any suitable area it can find which can include tree roots, hollow logs, or abandoned burrows of other species.

The Least Weasel is a diurnal predator and its diet is made up of small mammals (mostly rodents) but can also include birds’ eggs, lizards, frogs, salamanders, fish, worms and carrion ( Sheffield & King, 1994). It consumes a third of its body weight each day ( Woodland Trust, 2025). It also caches food to sustain it throughout the winter period ( Danzig, 1992). The distribution of rodents is a major factor in determining the habitat requirements of this species. It avoids open areas, when hunting, in an attempt to reduce the risk it faces from predation by birds of prey ( Sheffield & King, 1994).

*Mustela nivalis* was historically kept in homes to predate mice and small rodents ( Masseti, 1995) and has also been used for fur, food and traditional medicine in some areas of the world ( Lebarbenchon *et al.*, 2010). The main threats to this species currently include unintended ingestion of rodenticides, persecution and habitat loss due to changes in agriculture ( McDonald *et al.*, 2019; Sheffield & King, 1994).

Genome studies have been shown to be important when assessing introduced populations of this species to attempt to discern potential geographic origins of introduced populations, whether there was a single, or multiple introductions, and estimating when these introductions may have occurred ( Rodrigues *et al.*, 2017).

This species is classified as least concern by the IUCN Red list as it found over a wide distribution, its population is presumed to be large and unlikely to be declining overall although this is the case in some areas (for example, some areas of Britain Battersby, 2005). It is also found in a number of protected areas and shows tolerance to some degree of habitat modification ( McDonald *et al.*, 2019).

**Figure 1.**
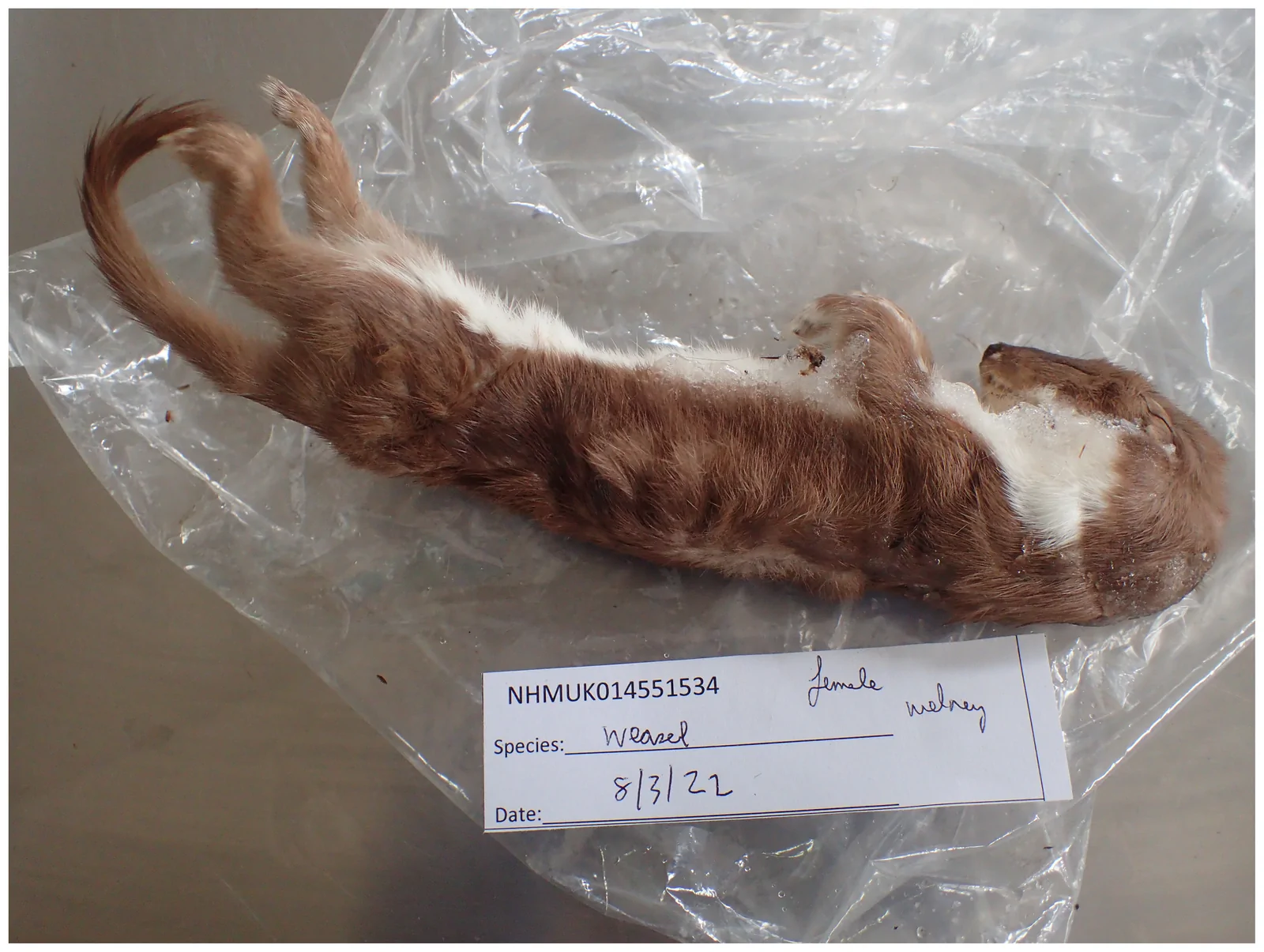
Photograph of the *Mustela nivalis* (mMusNiv2) specimen used for genome sequencing.

This genome was assembled using the Tree of Life pipeline from a specimen collected in Station Road, Welney, Cambridgeshire, England, United Kingdom ( Figure 1).

## Methods

### Sample acquisition

The specimen used for genome sequencing was an adult male *Mustela nivalis* (specimen ID NHMUK014551534, ToLID mMusNiv2; Figure 1), collected from Station Road, Welney, Cambridgeshire, England, United Kingdom (latitude 52.52, longitude −0.28) on 2022-03-08. The sample for this specimen was collected from a deceased wild individual that was euthanased as part of a pest control programme and stored at −20 °C. Muscle tissue samples were taken from areas of the thigh and the back. The specimen was collected and identified by Michelle O’Brien (Wildfowl & Wetlands Trust). Sample metadata was collected in line with the Darwin Tree of Life project standards described by Lawniczak *et al*. (2022).

### Nucleic acid extraction

Protocols for high molecular weight (HMW) DNA extraction developed at the Wellcome Sanger Institute (WSI) Tree of Life Core Laboratory are available on protocols.io ( Howard *et al.*, 2025). The mMusNiv2 sample was weighed and triaged to determine the appropriate extraction protocol. Tissue from the muscle was homogenised by cryogenic disruption using the Covaris cryoPREP ^®^ Automated Dry Pulverizer. HMW DNA was extracted using the Automated MagAttract v2 protocol. DNA was sheared into an average fragment size of 12–20 kb following the Megaruptor®3 for LI PacBio protocol. Sheared DNA was purified by automated SPRI (solid-phase reversible immobilisation). The concentration of the sheared and purified DNA was assessed using a Nanodrop spectrophotometer and Qubit Fluorometer using the Qubit dsDNA High Sensitivity Assay kit. Fragment size distribution was evaluated by running the sample on the FemtoPulse system. For this sample, the final post-shearing DNA had a Qubit concentration of 46.8 ng/μL and a yield of 6 084.00 ng. The 260/280 spectrophotometric ratio was 1.85, and the 260/230 ratio was 1.8.

RNA was extracted from muscle tissue of mMusNiv2 in the Tree of Life Laboratory at the WSI using the RNA Extraction: Automated MagMax™ *mir*Vana protocol. The RNA concentration was assessed using a Nanodrop spectrophotometer and a Qubit Fluorometer using the Qubit RNA Broad-Range Assay kit. Analysis of the integrity of the RNA was done using the Agilent RNA 6000 Pico Kit and Eukaryotic Total RNA assay.

### PacBio HiFi library preparation and sequencing

Library preparation and sequencing were performed at the WSI Scientific Operations core. Libraries were prepared using the SMRTbell Prep Kit 3.0 (Pacific Biosciences, California, USA), following the manufacturer’s instructions. The kit includes reagents for end repair/A-tailing, adapter ligation, post-ligation SMRTbell bead clean-up, and nuclease treatment. Size selection and clean-up were performed using diluted AMPure PB beads (Pacific Biosciences). DNA concentration was quantified using a Qubit Fluorometer v4.0 (ThermoFisher Scientific) and the Qubit 1X dsDNA HS assay kit. Final library fragment size was assessed with the Agilent Femto Pulse Automated Pulsed Field CE Instrument (Agilent Technologies) using the gDNA 55 kb BAC analysis kit.

The sample was sequenced on a Revio instrument (Pacific Biosciences). The prepared library was normalised to 2 nM, and 15 μL was used for making complexes. Primers were annealed and polymerases bound to generate circularised complexes, following the manufacturer’s instructions. Complexes were purified using 1.2X SMRTbell beads, then diluted to the Revio loading concentration (200–300 pM) and spiked with a Revio sequencing internal control. The sample was sequenced on a Revio 25M SMRT cell. The SMRT Link software (Pacific Biosciences), a web-based workflow manager, was used to configure and monitor the run and to carry out primary and secondary data analysis.

### Hi-C

***Sample preparation and crosslinking***

The Hi-C sample was prepared from 20–50 mg of frozen muscle tissue of the mMusNiv2 sample using the Arima-HiC v2 kit (Arima Genomics). Following the manufacturer’s instructions, tissue was fixed and DNA crosslinked using TC buffer to a final formaldehyde concentration of 2%. The tissue was homogenised using the Diagnocine Power Masher-II. Crosslinked DNA was digested with a restriction enzyme master mix, biotinylated, and ligated. Clean-up was performed with SPRISelect beads before library preparation. DNA concentration was measured with the Qubit Fluorometer (Thermo Fisher Scientific) and Qubit HS Assay Kit. The biotinylation percentage was estimated using the Arima-HiC v2 QC beads.

***Hi-C library preparation and sequencing***

Biotinylated DNA constructs were fragmented using a Covaris E220 sonicator and size selected to 400–600 bp using SPRISelect beads. DNA was enriched with Arima-HiC v2 kit Enrichment beads. End repair, A-tailing, and adapter ligation were carried out with the NEBNext Ultra II DNA Library Prep Kit (New England Biolabs), following a modified protocol where library preparation occurs while DNA remains bound to the Enrichment beads. Library amplification was performed using KAPA HiFi HotStart mix and a custom Unique Dual Index (UDI) barcode set (Integrated DNA Technologies). Depending on sample concentration and biotinylation percentage determined at the crosslinking stage, libraries were amplified with 10–16 PCR cycles. Post-PCR clean-up was performed with SPRISelect beads. Libraries were quantified using the AccuClear Ultra High Sensitivity dsDNA Standards Assay Kit (Biotium) and a FLUOstar Omega plate reader (BMG Labtech).

Prior to sequencing, libraries were normalised to 10 ng/μL. Normalised libraries were quantified again and equimolar and/or weighted 2.8 nM pools. Pool concentrations were checked using the Agilent 4200 TapeStation (Agilent) with High Sensitivity D500 reagents before sequencing. Sequencing was performed using paired-end 150 bp reads on the Illumina NovaSeq X.

### RNA library preparation and sequencing

Libraries were prepared using the NEBNext ^®^ Ultra™ II Directional RNA Library Prep Kit for Illumina (New England Biolabs), following the manufacturer’s instructions. Poly(A) mRNA in the total RNA solution was isolated using oligo(dT) beads, converted to cDNA, and uniquely indexed; 14 PCR cycles were performed. Libraries were size-selected to produce fragments between 100–300 bp. Libraries were quantified, normalised, pooled to a final concentration of 2.8 nM, and diluted to 150 pM for loading. Sequencing was carried out on the Illumina NovaSeq X to generate 150-bp paired-end reads.

### Genome assembly

Prior to assembly of the PacBio HiFi reads, a database of *k*-mer counts ( *k* = 31) was generated from the filtered reads using FastK. GenomeScope2 ( Ranallo-Benavidez *et al.*, 2020) was used to analyse the *k*-mer frequency distributions, providing estimates of genome size, heterozygosity, and repeat content.

The HiFi reads were assembled using Hifiasm in Hi-C phasing mode ( Cheng *et al.*, 2021; Cheng *et al.*, 2022), producing two haplotypes. Hi-C reads ( Rao *et al.*, 2014) were mapped to the primary contigs using bwa-mem2 ( Vasimuddin *et al.*, 2019). Contigs were further scaffolded with Hi-C data in YaHS ( Zhou *et al.*, 2023), using the --break option for handling potential misassemblies. The scaffolded assemblies were evaluated using Gfastats ( Formenti *et al.*, 2022), BUSCO ( Manni *et al.*, 2021) and MERQURY.FK ( Rhie *et al.*, 2020).

The mitochondrial genome was assembled using MitoHiFi ( Uliano-Silva *et al.*, 2023), which runs MitoFinder ( Allio *et al.*, 2020) and uses these annotations to select the final mitochondrial contig and to ensure the general quality of the sequence.

### Assembly curation

The assembly was decontaminated using the Assembly Screen for Cobionts and Contaminants ( ASCC) pipeline. TreeVal was used to generate the flat files and maps for use in curation. Manual curation was conducted primarily in PretextView and HiGlass ( Kerpedjiev *et al.*, 2018). Scaffolds were visually inspected and corrected as described by Howe *et al*. (2021). Manual corrections included 62 breaks and 188 joins. Chromosomes X and Y were identified by copy number in the diploid assembly. The curation process is documented at https://gitlab.com/wtsi-grit/rapid-curation. PretextSnapshot was used to generate a Hi-C contact map of the final assembly.

### Assembly quality assessment

The Merqury.FK tool ( Rhie *et al.*, 2020) was run in a Singularity container ( Kurtzer *et al.*, 2017) to evaluate *k*-mer completeness and assembly quality for both haplotypes using the *k*-mer databases ( *k* = 31) computed prior to genome assembly. The analysis outputs included assembly QV scores and completeness statistics.

The genome was analysed using the BlobToolKit pipeline, a Nextflow implementation of the earlier Snakemake version ( Challis *et al.*, 2020). The pipeline aligns PacBio reads using minimap2 ( Li, 2018) and SAMtools ( Danecek *et al.*, 2021) to generate coverage tracks. It runs BUSCO ( Manni *et al.*, 2021) using lineages identified from NCBI Taxonomy ( Schoch *et al.*, 2020). For the three domain-level lineages, BUSCO genes are aligned to the UniProt Reference Proteomes database ( Bateman *et al.*, 2023) using DIAMOND blastp ( Buchfink *et al.*, 2021).The genome is divided into chunks based on the density of BUSCO genes from the closest taxonomic lineage, and each chunk is aligned to the UniProt Reference Proteomes database with DIAMOND blastx. Sequences without hits are chunked using seqtk and aligned to the NT database with blastn ( Altschul *et al.*, 1990). The BlobToolKit suite consolidates all outputs into a blobdir for visualisation. The BlobToolKit pipeline was developed using nf-core tooling ( Ewels *et al.*, 2020) and MultiQC ( Ewels *et al.*, 2016), with package management via Conda and Bioconda ( Grüning *et al.*, 2018), and containerisation through Docker ( Merkel, 2014) and Singularity ( Kurtzer *et al.*, 2017).

## Genome sequence report

### Sequence data

**Figure 2.**
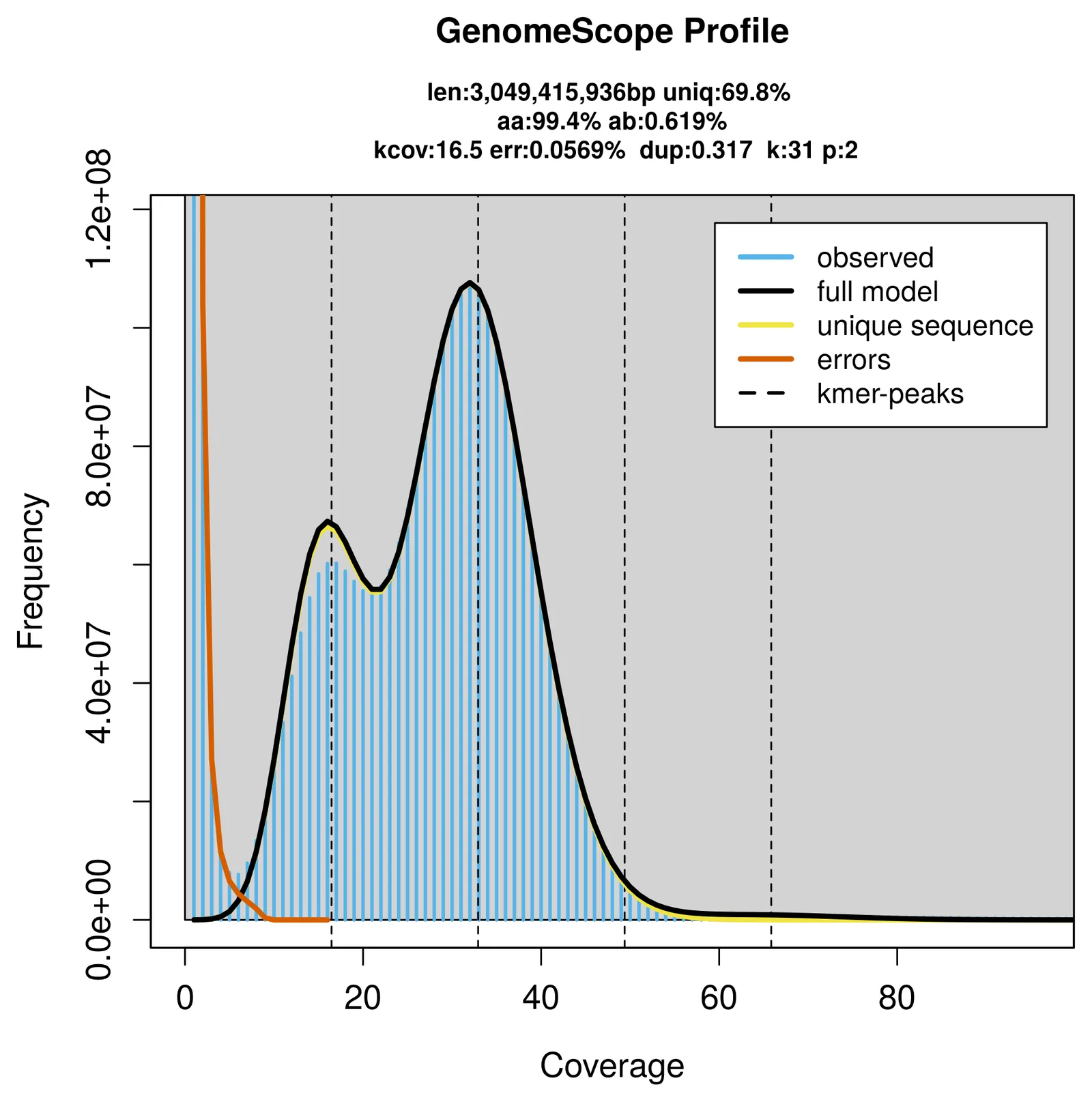
Frequency distribution of *k*-mers generated using GenomeScope2. The plot shows observed and modelled *k*-mer spectra, providing estimates of genome size, heterozygosity, and repeat content based on unassembled sequencing reads.

The genome of a specimen of *Mustela nivalis* was sequenced using Pacific Biosciences single-molecule HiFi long reads, generating 102.55 Gb (gigabases) from 12.08 million reads, which were used to assemble the genome. GenomeScope2.0 analysis estimated the haploid genome size at 3 049.42 Mb, with a heterozygosity of 0.62% and repeat content of 30.25% ( Figure 2). These estimates guided expectations for the assembly. Based on the estimated genome size, the sequencing data provided approximately 33× coverage. Hi-C sequencing produced 542.62 Gb from 3 593.52 million reads, which were used to scaffold the assembly. RNA sequencing data were also generated and are available in public sequence repositories. Table 1 summarises the specimen and sequencing details.

**Table 1.** Specimen and sequencing data for BioProject PRJEB80975.

Platform	PacBio HiFi	Hi-C	RNA-seq
**ToLID**	mMusNiv2	mMusNiv2	mMusNiv2
**Specimen ID**	NHMUK014551534	NHMUK014551534	NHMUK014551534
**BioSample (source** **individual)**	SAMEA113398841	SAMEA113398841	SAMEA113398841
**BioSample (tissue)**	SAMEA113398902	SAMEA113398902	SAMEA113398902
**Tissue**	muscle	muscle	muscle
**Instrument**	Revio	Illumina NovaSeq X	Illumina NovaSeq X
**Run accessions**	ERR13800511; ERR13800510	ERR13802650	ERR13802651
**Read count total**	12.08 million	3 593.52 million	61.72 million
**Base count total**	102.55 Gb	542.62 Gb	9.32 Gb

### Assembly statistics

The genome was assembled into two haplotypes using Hi-C phasing. Haplotype 1 was curated to chromosome level, while haplotype 2 was assembled to scaffold level. The final assembly has a total length of 3 437.86 Mb in 3 179 scaffolds, with 2 938 gaps, and a scaffold N50 of 115.39 Mb ( Table 2).

**Table 2.** Genome assembly statistics.

**Assembly name**	mMusNiv2.hap1.1	mMusNiv2.hap2.1
**Assembly accession**	GCA_964662115.1	GCA_964662095.1
**Assembly level**	chromosome	scaffold
**Span (Mb)**	3 437.86	2 835.05
**Number of** ** chromosomes**	22	N/A
**Number of contigs**	6 117	4 948
**Contig N50**	1.16 Mb	1.18 Mb
**Number of scaffolds**	3 179	2 239
**Scaffold N50**	115.39 Mb	121.45 Mb
**Longest scaffold length** ** (Mb)**	215.72	N/A
**Sex chromosomes**	X and Y	N/A
**Organelles**	Mitochondrial genome: 16.49 kb	N/A

**Figure 3.**
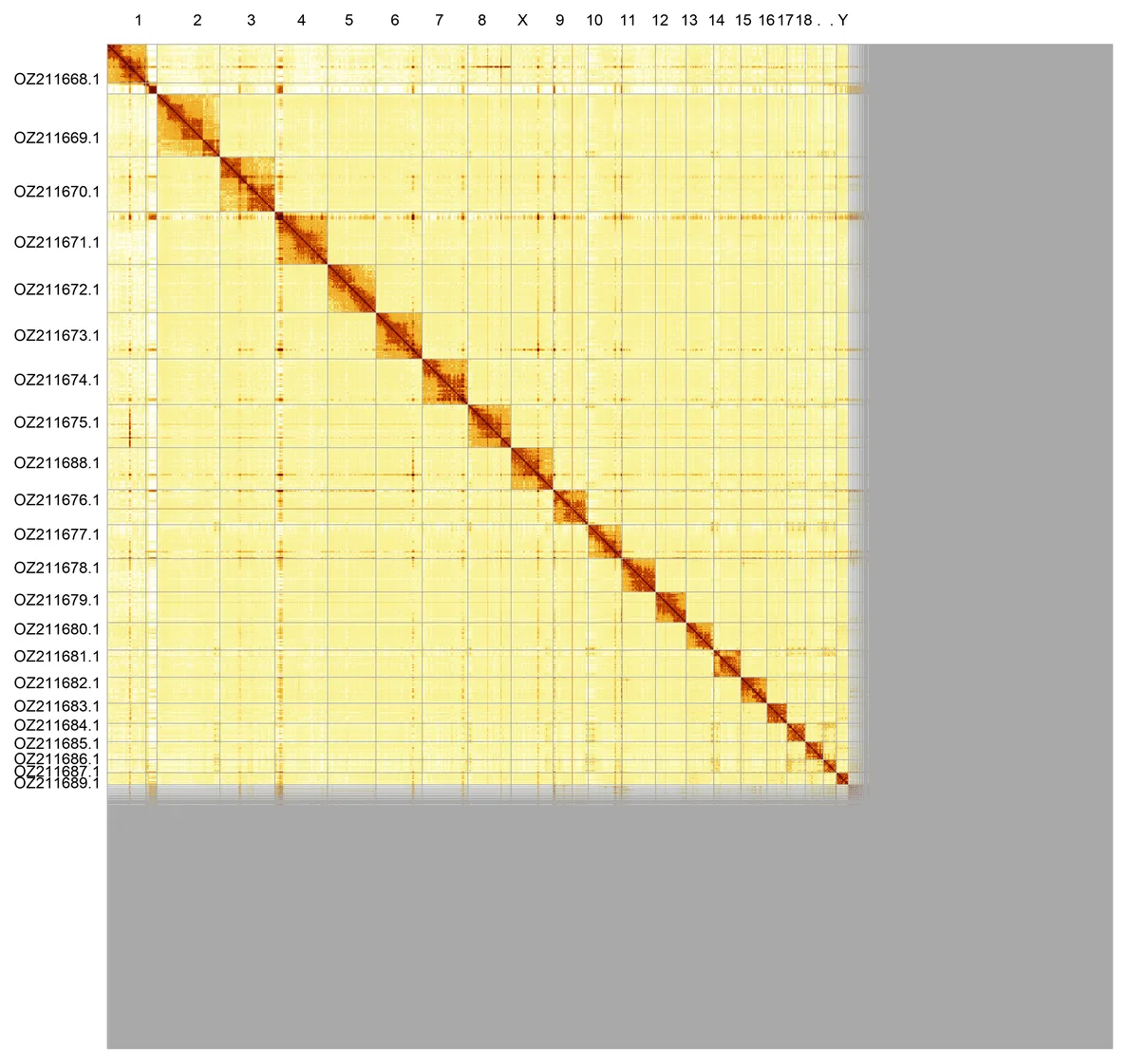
Hi-C contact map of the *Mustela nivalis* genome assembly. Assembled chromosomes are shown in order of size and labelled along the axes, with a megabase scale shown below. The plot was generated using PretextSnapshot.

Most of the assembly sequence (73.71%) was assigned to 22 chromosomal-level scaffolds, representing 20 autosomes and the X and Y sex chromosomes. These chromosome-level scaffolds, confirmed by Hi-C data, are named according to size ( Figure 3; Table 3). The mitochondrial genome was also assembled. This sequence is included as a contig in the multifasta file of the genome submission and as a standalone record.

**Table 3.** Chromosomal pseudomolecules in the haplotype 1 genome assembly of *Mustela nivalis* mMusNiv2.

INSDC accession	Molecule	Length (Mb)	GC%
OZ211668.1	1	215.72	41
OZ211669.1	2	187.21	41.50
OZ211670.1	3	180.52	40.50
OZ211671.1	4	165.13	38.50
OZ211672.1	5	158.07	40
OZ211673.1	6	156.19	41.50
OZ211674.1	7	147.41	41.50
OZ211675.1	8	143.66	43
OZ211676.1	9	120.18	43
OZ211677.1	10	115.39	43
OZ211678.1	11	114.20	40
OZ211679.1	12	105.23	40.50
OZ211680.1	13	93.33	43
OZ211681.1	14	92.85	43
OZ211682.1	15	89.49	39.50
OZ211683.1	16	68.89	43.50
OZ211684.1	17	62.33	47
OZ211685.1	18	61.79	46
OZ211686.1	19	44.26	48
OZ211687.1	20	41.63	41
OZ211688.1	X	133.52	40.50
OZ211689.1	Y	37.15	44.50
OZ211690.1	MT	0.02	40

### Assembly quality metrics

**Figure 4.**
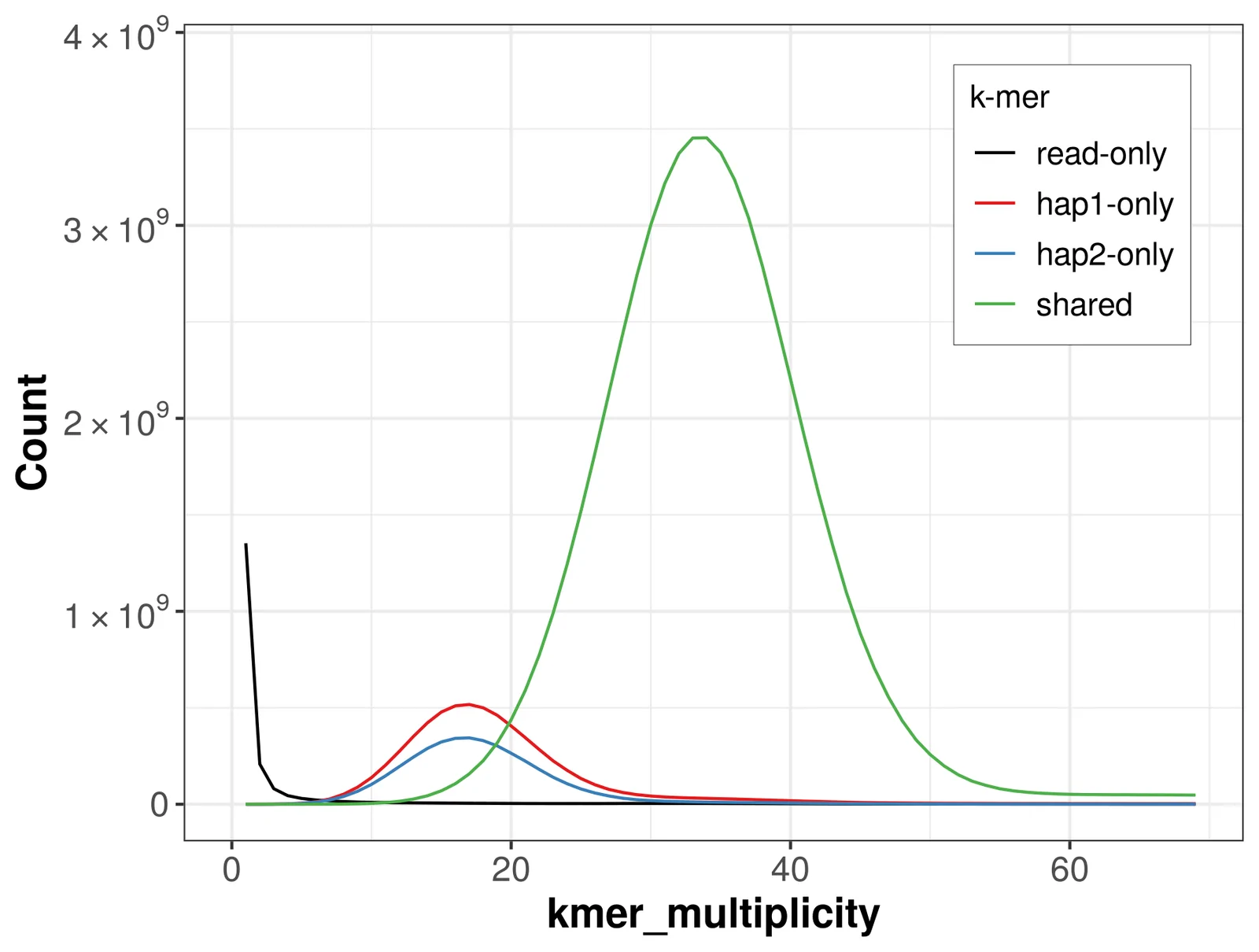
Evaluation of *k*-mer completeness using MerquryFK. This plot illustrates the recovery of *k*-mers from the original read data in the final assemblies. The horizontal axis represents *k*-mer multiplicity, and the vertical axis shows the number of *k*-mers. The black curve represents *k*-mers that appear in the reads but are not assembled. The green curve (the homozygous peak) corresponds to *k*-mers shared by both haplotypes and the red and blue curves (the heterozygous peaks) show *k*-mers found only in one of the haplotypes.

**Figure 5.**
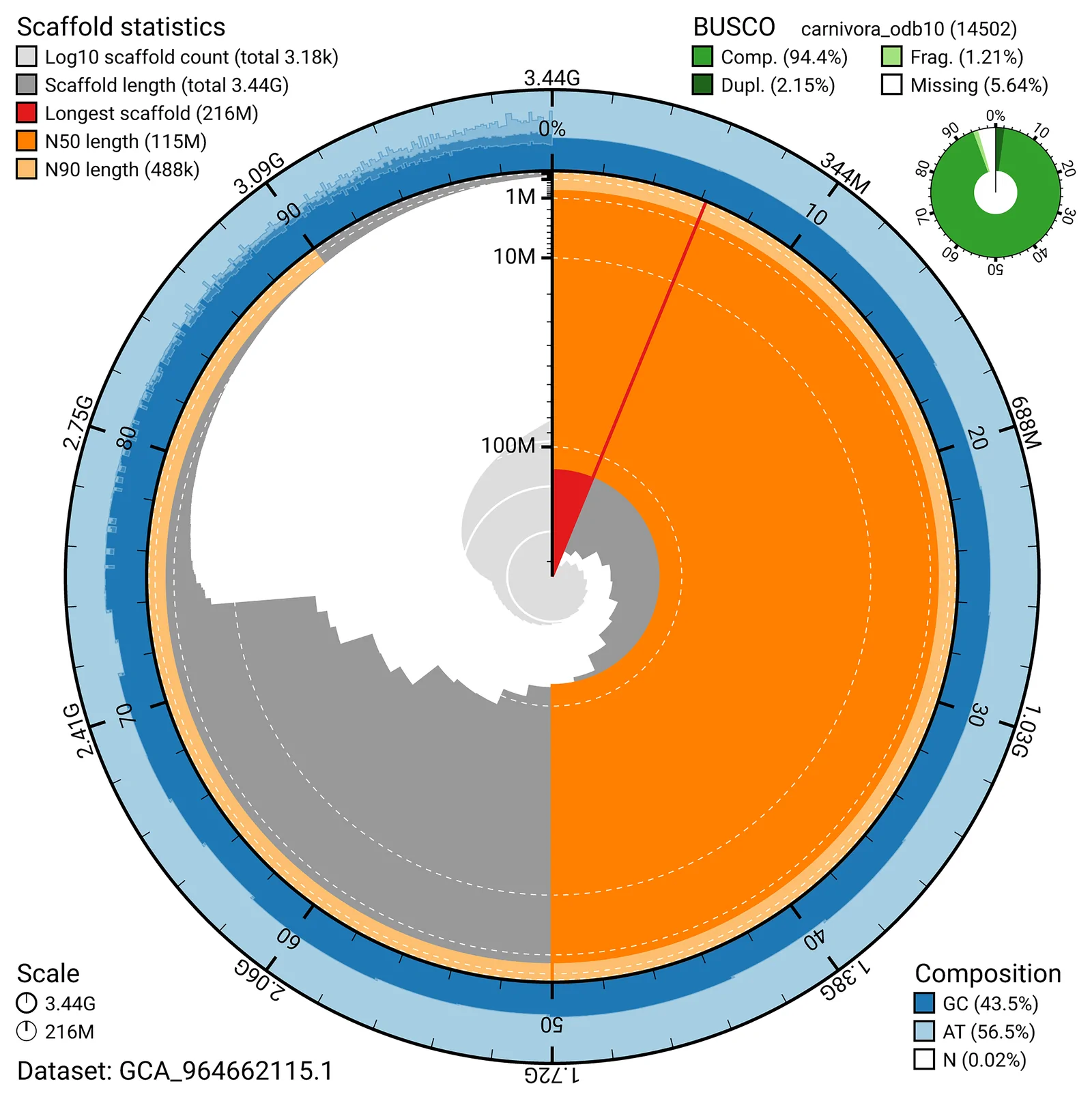
Assembly metrics for mMusNiv2.hap1.1. The BlobToolKit snail plot provides an overview of assembly metrics and BUSCO gene completeness. The circumference represents the length of the whole genome sequence, and the main plot is divided into 1 000 bins around the circumference. The outermost blue tracks display the distribution of GC, AT, and N percentages across the bins. Scaffolds are arranged clockwise from longest to shortest and are depicted in dark grey. The longest scaffold is indicated by the red arc, and the deeper orange and pale orange arcs represent the N50 and N90 lengths. A light grey spiral at the centre shows the cumulative scaffold count on a logarithmic scale. A summary of complete, fragmented, duplicated, and missing BUSCO genes in the set is presented at the top right. An interactive version of this figure can be accessed on the BlobToolKit viewer.

**Figure 6.**
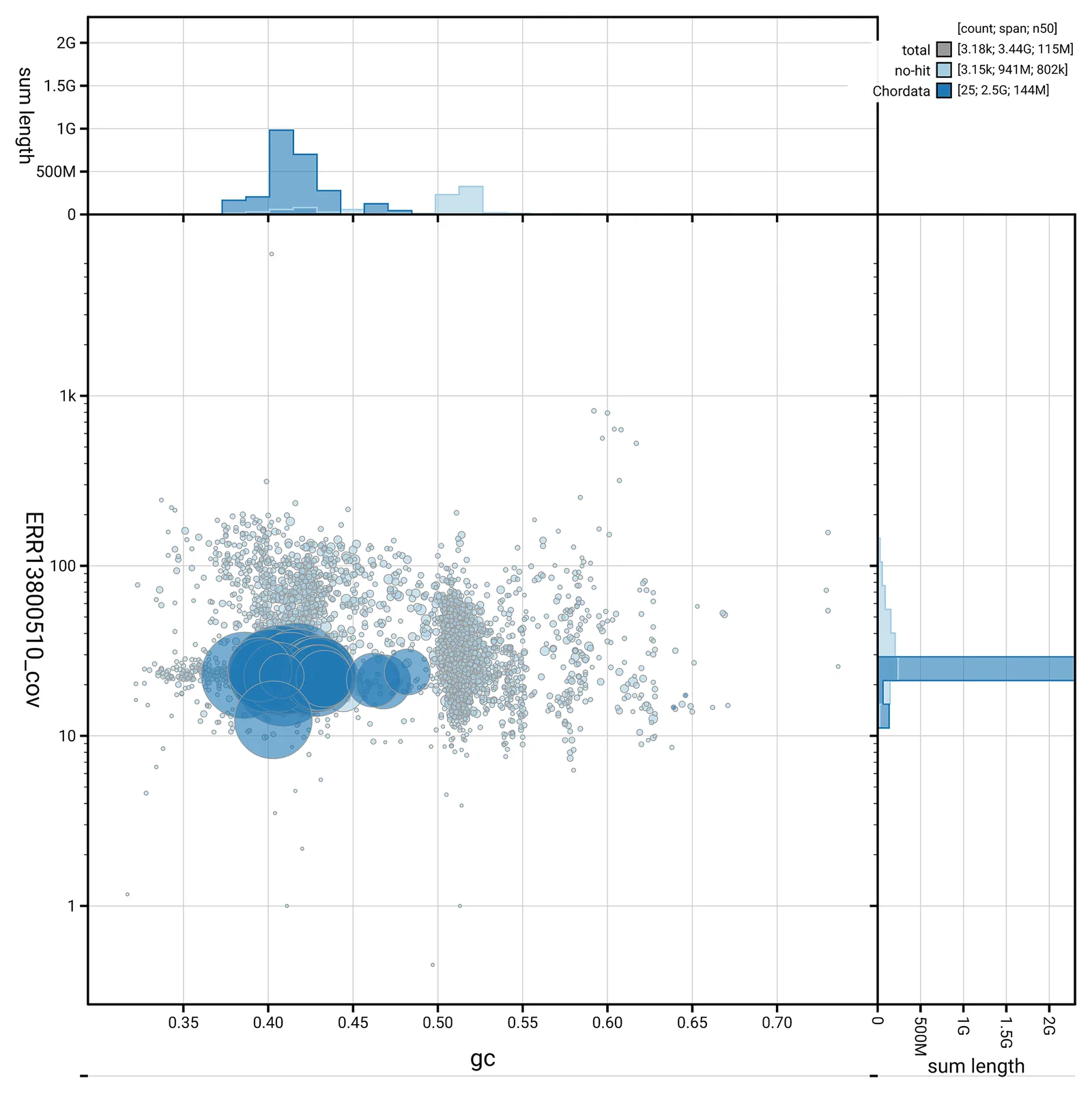
BlobToolKit GC-coverage plot for mMusNiv2.hap1.1. Blob plot showing sequence coverage (vertical axis) and GC content (horizontal axis). The circles represent scaffolds, with the size proportional to scaffold length and the colour representing phylum membership. The histograms along the axes display the total length of sequences distributed across different levels of coverage and GC content. An interactive version of this figure is available on the BlobToolKit viewer.

For haplotype 1, the estimated QV is 64.1, and for haplotype 2, 64.1. When the two haplotypes are combined, the assembly achieves an estimated QV of 64.1. The *k*-mer completeness is 89.43% for haplotype 1, 84.68% for haplotype 2, and 99.43% for the combined haplotypes ( Figure 4). BUSCO analysis using the carnivora_odb10 reference set ( *n* = 14 502) identified % of the expected gene set (single = %, duplicated = %) for haplotype 1. The snail plot in Figure 5 summarises the scaffold length distribution and other assembly statistics for haplotype 1. The blob plot in Figure 6 shows the distribution of scaffolds by GC proportion and coverage for haplotype 1.

Table 4 lists the assembly metric benchmarks adapted from Rhie *et al.* (2021) the Earth BioGenome Project Report on Assembly Standards September 2024. The EBP metric, calculated for the haplotype 1, is **6.8.Q64**.

**Table 4.** Earth Biogenome Project summary metrics for the *Mustela nivalis* assembly.

Measure	Value	Benchmark
EBP summary (haplotype 1)	6.8.Q64	6.C.Q40
Contig N50 length	1.16 Mb	≥ 1 Mb
Scaffold N50 length	115.39 Mb	= chromosome N50
Consensus quality (QV)	Haplotype 1: 64.1; haplotype 2: 64.1; combined: 64.1	≥ 40
*k*-mer completeness	Haplotype 1: 89.43%; Haplotype 2: 84.68%; combined: 99.43%	≥ 95%
BUSCO	C:94.4%[S:92.2%;D:2.2%]; F:1.2%; M:4.4%; n:14 502	S > 90%; D < 5%
Percentage of assembly assigned to chromosomes	73.71%	≥ 90%

### Wellcome Sanger Institute – Legal and Governance

The materials that have contributed to this genome note have been supplied by a Darwin Tree of Life Partner. The submission of materials by a Darwin Tree of Life Partner is subject to the **‘Darwin Tree of Life Project Sampling Code of Practice’**, which can be found in full on the [Darwin Tree of Life website] ( https://www.darwintreeoflife.org/project-resources/). By agreeing with and signing up to the Sampling Code of Practice, the Darwin Tree of Life Partner agrees they will meet the legal and ethical requirements and standards set out within this document in respect of all samples acquired for, and supplied to, the Darwin Tree of Life Project. Further, the Wellcome Sanger Institute employs a process whereby due diligence is carried out proportionate to the nature of the materials themselves, and the circumstances under which they have been/are to be collected and provided for use. The purpose of this is to address and mitigate any potential legal and/or ethical implications of receipt and use of the materials as part of the research project, and to ensure that in doing so we align with best practice wherever possible. The overarching areas of consideration are:

- Ethical review of provenance and sourcing of the material
- Legality of collection, transfer and use (national and international)

Each transfer of samples is further undertaken according to a Research Collaboration Agreement or Material Transfer Agreement entered into by the Darwin Tree of Life Partner, Genome Research Limited (operating as the Wellcome Sanger Institute), and in some circumstances, other Darwin Tree of Life collaborators.

## Data Availability

European Nucleotide Archive: Mustela nivalis (least weasel). Accession number PRJEB80975. The genome sequence is released openly for reuse. The *Mustela nivalis* genome sequencing initiative is part of the Darwin Tree of Life Project (PRJEB40665), Sanger Institute Tree of Life Programme (PRJEB43745) and Vertebrate Genomes Project (PRJNA489243). All raw sequence data and the assembly have been deposited in INSDC databases. The genome will be annotated using available RNA-Seq data and presented through the Ensembl pipeline at the European Bioinformatics Institute. Raw data and assembly accession identifiers are reported in Table 1 and Table 2. Pipelines used for genome assembly at the WSI Tree of Life are available at https://pipelines.tol.sanger.ac.uk/pipelines. Table 5 lists software versions used in this study.

## Author information

- Members of the Natural History Museum Genome Acquisition Lab
- Members of the Wellcome Sanger Institute Tree of Life Management, Samples and Laboratory team
- Members of Wellcome Sanger Institute Scientific Operations – Sequencing Operations
- Members of the Wellcome Sanger Institute Tree of Life Core Informatics team
- Members of the Tree of Life Core Informatics collective
- Members of the Darwin Tree of Life Consortium
