# Supplementary material for: The genome sequence of the least weasel, Mustela nivalis Linnaeus, 1766 (Carnivora: Mustelidae)
Source: Wellcome Open Res. 2025 Sep 8;10:488. [Version 1] doi: 10.12688/wellcomeopenres.24862.1 (PMC13589180; doi:10.12688/wellcomeopenres.24862.1)
Supplement: Peer review history

# Reviewer report: Reviewer response for version 1

**DOI:** 10.21956/wellcomeopenres.27386.r136643

**Published:** 2025-10-31

**Authors:** Manuel Ruedi

**Affiliations:** 1 Mammalogy, Museum d'histoire naturelle de la Ville de Geneve, Geneva, Geneva, Switzerland; 2 Université de Genève, Genève, Switzerland

The current version of the manuscript is excellent and clear, and I have only the following suggestion:

 regarding colouration: it would be interesting to note that some subspecies (or colouration morphs) of least weasel turn white in winter, others not, due to a single adaptive amino acid mutation, as shown in a recent museomics study [(Miranda I, et al., 2021) - ref 1]. In England (as far as I known), the least weasel does not change coat coloration and keeps brown fur year-round and thus should belong to the “vulgaris” morph (verify in its genome).

Are sufficient details of methods and materials provided to allow replication by others?

Yes

Is the rationale for creating the dataset(s) clearly described?

Yes

Are the datasets clearly presented in a useable and accessible format?

Yes

Are the protocols appropriate and is the work technically sound?

Yes

Reviewer Expertise:

Systematics and zoology

I confirm that I have read this submission and believe that I have an appropriate level of expertise to confirm that it is of an acceptable scientific standard.

## References

[1] : Museomics Dissects the Genetic Basis for Adaptive Seasonal Coloration in the Least Weasel. *Molecular Biology and Evolution* .2021;38(10) : 10.1093/molbev/msab1774388-4402 10.1093/molbev/msab177 PMC847613334157721

# Reviewer report: Reviewer response for version 1

**DOI:** 10.21956/wellcomeopenres.27386.r133798

**Published:** 2025-10-07

**Authors:** Edgar G Gutierrez

**Affiliations:** 1 National Polytechnic Institute, México, Santo Tomás, Mexico

The manuscript provides an important genomic resource and is overall well written. However, several sections would benefit from clarification, improved conciseness, and greater consistency in formatting and presentation. Addressing the following points will strengthen the clarity and impact of the work.

- Please clarify whether ecological traits (e.g., solitary nature, breeding season and the birth of the pups, pest status) occur across the species’ entire distribution or only in specific regions.
- When discussing introduced populations, provide more context: which populations are non-native, and why genomic studies are especially relevant to evaluate them.
- The rationale for generating two haplotypes with different assembly levels. (chromosome- vs scaffold-level) should be explained. Why was only one haplotype analyzed with BlobToolKit? Including results for the second haplotype (e.g., as supplementary material) would be valuable.
- The section on sequencing with the Revio platform is somewhat redundant. Please simplify and present details more concisely.
- The RNA-seq library preparation section also repeats phrasing (e.g., “Libraries were…”). Consider merging steps (quantification, pooling, normalization) into integrated sentences for readability.
- The BlobToolKit pipeline description is dense, with many tools listed consecutively. Consider splitting into two sentences or rephrasing to emphasize workflow logic.
- Figure 1: while the specimen photo is accurate, a more engaging image (e.g., a wild individual with permissions) could improve presentation.
- Provide units/format for the coordinates; is this notation standard?
- Italicize the scientific name throughout the text (e.g., *Mustela nivalis*).
- Define Mb (megabases) at first use, as done with Gb.
- Specify the BAC analysis kit manufacturer (e.g., Agilent Technologies).
- Correct typographical issues, such as missing spaces after periods (e.g., “…blastp…).The genome…”).

Are sufficient details of methods and materials provided to allow replication by others?

Yes

Is the rationale for creating the dataset(s) clearly described?

Yes

Are the datasets clearly presented in a useable and accessible format?

Partly

Are the protocols appropriate and is the work technically sound?

Yes

Reviewer Expertise:

genomics, immunogenetics, evolutionary biology

I confirm that I have read this submission and believe that I have an appropriate level of expertise to confirm that it is of an acceptable scientific standard, however I have significant reservations, as outlined above.
